# Attitudes and barriers to resistance exercise training for older adults living with multiple long-term conditions, frailty, and a recent deterioration in health: qualitative findings from the Lifestyle in Later Life – Older People’s Medicine (LiLL-OPM) study

**DOI:** 10.1186/s12877-023-04461-5

**Published:** 2023-11-24

**Authors:** Christopher Hurst, Lorelle Dismore, Antoneta Granic, Ellen Tullo, Jane M. Noble, Susan J. Hillman, Miles D. Witham, Avan A. Sayer, Richard M. Dodds, Sian M. Robinson

**Affiliations:** 1 AGE Research Group, Translational and Clinical Research Institute, Faculty of Medical Sciences, Newcastle University, Newcastle upon Tyne, UK; 2 NIHR Newcastle Biomedical Research Centre, Newcastle University, Newcastle upon Tyne NHS Foundation Trust and Cumbria, Northumberland, Tyne and Wear NHS Foundation Trust, Newcastle upon Tyne, UK; 3 Northumbria Healthcare NHS Foundation Trust, North Tyneside Hospital, Rake Lane, North Shields, Tyne & Wear, Newcastle upon Tyne, UK; 4 Department of Older People’s Medicine, Newcastle upon Tyne Hospitals NHS Foundation Trust, Newcastle upon Tyne, UK

**Keywords:** Resistance exercise, Frailty, Multimorbidity, Sarcopenia, Muscle strength, Psychosocial, Qualitative research, Perceptions

## Abstract

**Background:**

Many older adults live with the combination of multiple long-term conditions (MLTC) and frailty and are at increased risk of a deterioration in health requiring interaction with healthcare services. Low skeletal muscle strength is observed in individuals living with MLTC and is central to physical frailty. Resistance exercise (RE) is the best available treatment for improving muscle strength, but little is known about the attitudes and barriers to RE in this group of older adults. This study therefore aimed to explore the knowledge of and attitudes towards RE, as well as the barriers and enabling factors, in older adults living with MLTC, frailty and a recent deterioration in health.

**Methods:**

Fourteen participants aged 69–92 years (10 women) from the Lifestyle in Later Life – Older People’s Medicine (LiLL-OPM) study were recruited from an Older People’s Medicine Day Unit in Newcastle, UK. Participants were invited to take part in a semi-structured interview exploring their knowledge and attitudes as well as barriers and enabling factors to RE. Data were analysed using thematic analysis.

**Results:**

The analysis generated three themes (1) a lack of awareness and understanding of RE, (2) a self-perceived inability to perform RE; physical and psychological barriers and (3) willingness to perform RE under expert guidance. There was a general lack of awareness and understanding of RE, with most participants having never heard of the term and being unaware of its potential benefits. When RE was described, participants stated that they would be willing to try RE, but it was apparent that an individualised approach underpinned by expert guidance would be required to support engagement.

**Conclusions:**

Older adults living with MLTC, frailty and a recent deterioration in health lack awareness and understanding of RE. Despite a range of barriers, this group appear willing to engage in RE if they are appropriately supported. There is a need to co-design and deliver effective strategies, including education, to raise awareness and understanding of RE, as well as promote engagement in RE, in this group of older adults.

**Supplementary Information:**

The online version contains supplementary material available at 10.1186/s12877-023-04461-5.

## Introduction

A substantial number of older people live with multiple long-term conditions (MLTC or multimorbidity), defined as the presence of two or more long-term health conditions [1, 2]. The presence of MLTC is a major contributor to the onset and progression of frailty syndrome (a multi-system impairment associated with increased vulnerability to stressors [3]) with most older adults living with frailty being multimorbid [4]. These syndromes are complex, and the coexistence of MLTC and frailty interact to increase the risk of adverse outcomes beyond individual effects [3, 5, 6]. Older people living with MLTC and frailty are more likely to experience a deterioration in their health that requires interaction with healthcare services (such as specialist referral and/or hospital admission) than individuals without these syndromes [3]. Accordingly, this group of older adults are commonly seen within clinical practice [7], yet there remains a limited evidence base for their care as they are typically underserved by research [8, 9].

Low skeletal muscle strength is observed in individuals living with MLTC [10, 11] and is central to the physical phenotype of frailty [12]. Resistance exercise (RE) training is currently recommended as the first-line treatment for improving muscle strength and function in older people with frailty and guidance exists for clinicians and practitioners to support the delivery of effective programmes [13–15].

The exercise behaviour of older people is influenced by a range of diverse factors from multiple domains [16]. Several barriers to RE have been previously documented including individual (e.g., poor health, pain, fear of injury), psychological (e.g., attitudes and health beliefs), social (e.g., lack of social support) and environmental factors (e.g., lack of appropriate programmes and facilities) [17–20]. While there is less understanding of the specific factors that influence the exercise behaviour of older adults living with MLTC and frailty, particularly those with a recent deterioration in health, it is clear that behaviour in this group is influenced by a much wider and more complex range of factors [21, 22]. For example, the presence of MLTC may induce specific barriers to successful engagement in RE because of health-related factors such as increased treatment burden, pain, breathlessness, and fatigue [21]. These health-related factors may be increasingly pertinent in those who have experienced a recent decline in their health status. Conversely however, it may be that a deterioration in health acts as a motivating factor to engage in exercise [17]. In the specific context of RE there remains little understanding of the influences relevant to older people living with the combination of MLTC, frailty, and a recent deterioration in health who could benefit substantially from this exercise mode.

Despite growing evidence supporting the potential of RE to improve muscle strength and function for older people living with MLTC and frailty, there remains a need to understand more about how to co-design and deliver RE programmes that this group are willing and able to engage with. This study aimed to explore the knowledge of, and attitudes towards RE as well as the barriers and enabling factors to RE in a group of older adults living with MLTC, frailty and a recent deterioration in health.

## Method

### Study design

This qualitative investigation was part of the Lifestyle in Later Life – Older People’s Medicine (LiLL-OPM) study conducted in Newcastle upon Tyne, UK. A full description of the LiLL-OPM study including aims, recruitment strategy and data collection can be found in the published protocol paper [8]. Briefly, the LiLL-OPM study was designed to: 1) determine if it is feasible and acceptable to carry out a research project with older adults living with MLTC, frailty and a recent deterioration in health (identified as an illness.

episode requiring interaction with healthcare services) and 2) describe the health and lifestyle of these older adults, and included both quantitative and qualitative data collection [8]. The study included questionnaire-based assessment of several health and lifestyle factors including health status and physical function, physical activity, diet, appetite, smoking, and alcohol consumption. Frailty status was quantified using the Fried frailty score [12] and participants were asked to self-report the presence of long-term health conditions. The SARC-F (Strength, Assistance in walking, Rise from a chair, Climbing stairs, and Falls) questionnaire was used as a screening tool for sarcopenia [23]. Participants were asked to wear a wrist-worn triaxial accelerometer for 7-days (GENEActiv® Original, ActivInsights Ltd, Cambridge, UK) to provide an objective assessment of physical activity. The qualitative component of the LiLL-OPM study involved semi-structured interviews focusing on (1) how to involve this group of older adults in research, (2) RE training and (3) experiences of taking part in research. This paper presents a subset of the qualitative data collected in the LiLL-OPM study that focused on RE.

Ethical approval for the LiLL-OPM study was granted by the National Health Service (NHS) Health Research Authority, London – Harrow Research Ethics Committee (Ref 20/LO/1243). All participants provided written informed consent and the study was conducted in accordance with the Declaration of Helsinki.

### Participants

Participants were recruited into the LiLL-OPM study from an Older People’s Medicine (OPM) Day Unit service in Newcastle Hospitals, UK. Patients are typically referred to this Day Unit for Comprehensive Geriatric Assessment (CGA), including physical and mental health, functional, social, and environmental dimensions, because of a recent deterioration in their health (e.g., a fall, or unexplained weight loss). Patients were invited to participate in the study if they were living in their own home and had experienced a recent deterioration in health, with a referral to the OPM Day Unit. Potential participants were provided with an information sheet and a brief explanation of the study by a clinician during their visit to the Day Unit. Following time to consider involvement, patients were contacted by a member of the research team to discuss participation in the study. Any older adults who the OPM clinician felt it was inappropriate to approach (e.g., those with moderate to severe dementia, or metastatic cancer with prognosis of only a few weeks) and those who were unable to provide informed consent were excluded. There were no specific age criteria for inclusion in the study, although patients attending the OPM clinic are typically aged over 65 years. There was no upper age limit for inclusion in the study.

### Interviews

Data collection for this study involved in-depth, semi-structured interviews led by an experienced health psychologist (LD). Interviews took place between June and November 2021. Participants were offered the option of completing the interview either via a telephone/video call or a home visit with the researcher and could elect to have an informal carer present. An interview guide, consisting of open-ended questions, was developed, and refined by two of the authors (CH, LD) based on previous work and published literature. The interview guide can be found in the supplementary material available with this article (Additional file 1). Interviews began with general questions around physical activity and exercise to provide broader context and initiate the discussion with the participant. Participants were then asked if they had heard the term ‘resistance exercise’ (RE). Once participants were given the opportunity to answer this question, the researcher explained the term ‘resistance exercise’ using standardised text. Following this explanation, questions focused on RE specifically and sought to understand participant’s perspectives of and attitudes towards RE, for example, ‘*Can you tell me what your thoughts are on resistance exercise?*’ and ‘*How would you feel about doing this kind of exercise?*’. Interviews were audio-recorded and deleted once transcribed verbatim. All participants were allocated a pseudonym.

### Data analysis

Data were analysed using reflexive thematic analysis (TA) whereby the researcher’s subjectivity is central to the analytical procedure. Meaning was therefore generated through interpretation of data, and saturation was subjective [23, 24]. Reflexive TA provides a rich and detailed, yet complex account of data. The data were analysed using an inductive approach with emergent themes grounded within the data. The six steps involved familiarisation with the dataset by reading and re-reading the data, to become immersed with its content. Identification of interesting aspects of the data relevant to the research question were documented using codes. This involved highlighting text (short segments of the data) throughout the data transcripts and coding as much data as possible to represent meaning and patterns within the data. Initial themes were generated by examining the codes and collating data to develop significant broader patterns of meaning. Themes were developed and reviewed by checking the themes against the coded data and entire dataset. Themes were defined as patterns of shared meaning underpinned by a central concept or idea. Themes were defined, refined, and named between two authors (LD and CH) and finally, written up with supporting quotations. Data were managed and coded manually using Microsoft Word.

## Results

Fourteen participants (10 women, 4 men) from the LiLL-OPM study were invited to take part in a semi-structured interview. None of the patients who were invited declined participation. Interviews lasted 35 ± 10 (mean ± standard deviation [SD]) minutes with most interviews (12/14) taking place in participants’ own homes. Two participants were interviewed via telephone (n = 1) or video call (n = 1) and one participant’s informal carer was present during the interview. Participants were aged between 69 and 92 years (mean ± SD; 82 ± 7 years). Based on the Fried frailty criteria, 13 (93%) participants were pre-frail (n = 3) or frail (n = 10). Twelve participants (86%) were living with MLTC. All participants had recently experienced a decline in their health status as the reason for referral to the Older People’s Medicine Day Unit. The characteristics of the sample are summarised in Table 1.

**Table 1:** Characteristics of the sample

	All (n = 14)	Men (n = 4)	Women (n = 10)
Age (years)	82 (7)	84 (7)	81 (7)
Ethnicity			
White British	13 (93)	4 (100)	9 (90)
Asian or Asian British – Indian	1 (7)	0 (0)	1 (10)
Number of long-term conditions			
0–1 (No MLTC)	2 (14)	2 (50)	0 (0)
≥ 2 (MLTC)	12 (86)	2 (50)	10 (100)
Number of medications			
0–4	3 (21)	1 (25)	2 (20)
≥ 5	11 (79)	3 (75)	8 (80)
Fried frailty score			
0 (Non-frail)	1 (7)	1 (25)	0 (0)
1–2 (Pre-frail)	3 (21)	0 (0)	3 (30)
3+ (Frail)	10 (71)	3 (75)	7 (70)
SARC-F			
0	1 (7)	1 (25)	0 (0)
1	1 (7)	0 (0)	1 (10)
2	1 (7)	0 (0)	1 (10)
3	1 (7)	0 (0)	1 (10)
4+	10 (71)	3 (75)	7 (70)
Accommodation status			
Standard housing	11 (79)	3 (75)	8 (80)
Sheltered housing with warden	2 (14)	1 (25)	1 (10)
Assisted living (extra care)	1 (7)	0 (0)	1 (10)
Physical activity			
Mean acceleration (m*g*)*	13.79 [4.11]	10.53 [2.71]	15.24 [3.86]

Values shown are mean [standard deviation; SD] or count (%)

SARC-F: Strength, Assistance, Rise, Climb – Falls questionnaire. Simplified Questionnaire to Rapidly Diagnose Sarcopenia

* n = 13 (one participant [F] declined the physical activity assessment)

The thematic analysis generated three themes:1) a lack of awareness and understanding of RE, 2) a self-perceived inability to perform RE; physical and psychological barriers and 3) willingness to perform RE under expert guidance. Direct quotations are presented within the text to illustrate our findings (Sex, ID number).

### Theme 1: a lack of awareness and understanding of resistance exercise

There was a general lack of awareness and understanding of RE, with most participants having never heard of the term and being unaware of its potential benefits.

“*No, what is it?”* (Female, Aged 90).

*“I don’t know what exactly it means…I’m not sure if I am up to all that you know”* (Female, Aged 92).

*“It wouldn’t do you any good to lift heavy things, they tell you that, don’t lift heavy things you know, if you read through those magazines it tells you things like that”* (Female, Aged 77).

Participants reported their own individual interpretations and preferences for physical activity and exercise. For example, only one participant performed structured exercise, whilst others viewed exercise as activities such as housework, gardening, and daily routine chores. Participants described performing adequate exercise, and resistance exercise as unnecessary.

*“I’m just happy to go to the physiotherapy, I don’t want to get into too much, I really don’t, I mean I do everything for myself in this house, don’t get any help at all so I do a lot of exercise with that”* (Female, Aged 81).

*“I think the amount of exercise I get and the workings I do, I think is far greater than what I would get going to a gym for half an hour twice a week…and much heavier than what they would let me do at a gym I would think”* (Male, Aged 86).

Age-associated limitations were linked with the lack of perceived benefits of resistance exercise and that resistance exercise would be more suited to younger adults living without long-term conditions.

*“I don’t know if there would be any effects, maybe it would have an effect on a younger person not having arthritis”* (Female, Aged 92).

*“When you’re younger its fine but as you get older it’s just pain literally everywhere whatever you do, I can’t pick up anything without dropping it”* (Female, Aged 78).

Despite the lack of awareness and understanding of resistance exercise, most participants had previously received physiotherapy and used resistance bands to help with their physical health.

*“She’s giving me plastic thing [resistance band]…I think they do some benefit for me because you see I can work from my foot up to here and then I can pull like that with whatever she’s given me…I think she thought if I do that sort of exercises, so it would make me…more useful and more active”* (Female, Aged 69).

The participants emphasised the importance of the benefits of physiotherapy to help with their disability and in maintaining their independence. For some, they described a preference for continuing with physiotherapy as opposed to engaging in any further exercise.

### **Theme 2: a self-perceived inability to perform resistance exercise; physical and psychological barriers**

Physical and psychological barriers were emphasised as factors contributing to participants self-perceived inability to perform RE. Some of the participants felt that their age was associated with their inability to do resistance exercise.

*“I’m too old to be exercising, that’s the way I feel, I haven’t got the strength in us…I couldn’t go and lift weights”* (Female, Aged 86).

This was explained as having a perceived lack of strength to perform RE and a decline in physical strength due to ageing. This was associated with the lack of awareness and understanding of the benefits of RE.

*“Where are you going to get the strength to do that? Pushing against something…you just get weaker and weaker, don’t you? As you get older, you’ve got no push in you”* (Male, Aged 87)

*“I’ve got arthritis I have no strength in my hand because of my arthritis, I find it very difficult to push a button you know so I think I’m past all that… not weightlifting. Weightlifting I don’t want to try… I have never done it”* (Female, Aged 92).

Barriers to engagement were predominantly related to living with health conditions and experiencing disability including arthritis, pain, poor eyesight, and mobility issues.

*“There’s not a lot I can do really…because with that hand being like that and you’re holding on… my eyesight as well…because of the walking with this* [zimmer frame] *and just taking my time and everything is slow…I think I’m passed doing anything…”* Female, Aged 90)

Participants were concerned that they might cause themselves more damage, and they expressed a fear of falling due to balance issues and a fear of causing themselves an injury.

*“It worries us if I broke a finger and something, my hands are bad enough, but if something happened to them…if I injured myself… I’m fearful of hurting myself… sometimes if I go to do something, I think no I’m not doing that because I’m just going to fall and I’ll be on my own, because I’m on my own this is one of the big worries”* (Female, Aged 81).

*“I want to walk around and someone with me…because you see I’m a bit scared if I have a fall… I always want my husband with me…I feel like getting active again if I never had falls…I’m a bit scared”* (Female, Aged 69).

Other barriers to engaging in RE included a lack of motivation and a lack of energy to perform the exercises.

*“You need the motivation, mainly to do it, the lack of motivation and the lack of energy… you just don’t have that same incentive… bit more energy which I have none what’s so ever”* (Female, Aged 78).

These factors were associated with an unwillingness to increase activity levels or engage in RE.

### Theme 3: willingness to perform resistance exercise under expert guidance

Participants stated that they would be willing to try RE if they were advised to by a healthcare professional.

*“Yeah well, I probably would try”* (Female, Aged 81).

*“I would say okay I would give it a go”* (Female, Aged 81).

This advice would need to be supported by a personalised health assessment from a healthcare professional. Assessing their health conditions prior to prescribing RE would provide them with reassurance that it’s safe to perform the exercises.

*“If the doctor was saying to me ‘there’s nothing sinister going on and you’re fine, just try and get your body motivated’ then I would do that, but I’m frightened to do this in case I’m hurting something else… if he said it was all right for my bones, I would be saying ‘right I’ll give it a go’…I mean I’ve got to see the osteoporosis at the clinic…if they said it was all right* [physiotherapist advice], *yeah, I would do that”* (Female, Aged 79).

Some of the participants expressed the need for information about RE prior to commencing an exercise programme. For example, what this would entail before deciding on their engagement.

*“I just want to know what it’s about and if I found it was okay and helping I would do it, but I never say yes until I know what it involves… if I think it might be good, I’ll give it a go…if I benefit from it, well do it”* (Female, Aged 81).

Knowing that an exercise programme had been adjusted to their own individual health needs and having an understanding as to how the programme would personally benefit them, would encourage the older adults to perform the exercise.

*“It would be the doctor doing his work because I’m sure he will assess me beforehand…but I think if he was to look at everything going on within my body at the moment…and surely he would make an assessment before he would say that to me”* (Female, Aged 78).

From a practical perspective, some participants would be happy to exercise in a community centre whereas for others home-based exercise was a preference because of difficulties with transportation.

*‘Personally, I would do it at home, because it’s so complicated to get to a community centre or the hospital’* (Male, Aged 74).

For the older adults with mobility problems and a fear of falling they would need to feel supported when performing the exercise and for some, this meant a reliance on informal carers.

## Discussion

The aim of the present study was to explore knowledge and attitudes towards RE and develop an understanding of the barriers and facilitators to RE in older adults living with MLTC, frailty and a recent deterioration in health. We conducted semi-structured interviews to explore the perspectives and experiences of RE in a group of older adults who had been referred to an Older People’s Medicine clinic because of a recent deterioration in their health. Although RE has the potential to be an effective therapeutic strategy for this group of older adults, previous research has shown that few older people engage in structured RE and considerable barriers to engagement exist. As such, there remains a need to further understand these issues to effectively tailor interventions to meet the needs of this group.

We identified three key themes from our interviews. The first of these themes was a lack of awareness and understanding of RE. Despite several participants reporting that they had previously engaged in RE with a physiotherapist, most were not familiar with the term ‘resistance exercise’ (RE). This finding is consistent with previous work showing that older adults are generally unaware of RE and even those older adults who are physically active do not understand what RE is [20]. Some participants noted that they were already physically active and that engaging in RE would offer them no extra benefit—indicating a lack of understanding—a finding previously reported by Broderick et al. [22] who found that frail older adults viewed exercise as a by-product of another purposeful activity rather than an activity in itself. Lack of knowledge is a meaningful barrier to resistance exercise in older people [17] and increasing knowledge, including ‘*what is RE’*, ‘*how to perform RE’* and ‘*benefits of RE’*, could help to motivate individuals to engage [25]. As knowledge about an activity is an important determinant of behaviour [26], our findings highlight the need to build awareness and understanding of RE in older adults living with MLTC, frailty and a recent deterioration in health.

The second key theme from our data was a self-perceived inability to perform RE which was influenced by both physical and psychological barriers. Physical barriers including fatigue, poor health and risk of injury are commonly cited, individual-level barriers to exercise in older people [17]. These issues are likely to be exacerbated in those living with frailty, where exhaustion and weakness are central components of the frailty phenotype [12], while health problems such as pain, fatigue and lack of energy are substantial barriers to exercise in those living with MLTC [21]. Our participants identified a lack of strength as being a barrier to engaging in RE, yet a common motivator for older people to exercise is the physical health benefit of experiencing an increase in strength [17]. A fundamental aim of a RE programme is to increase muscle strength, yet our data illustrating a lack of awareness and understanding of RE (theme 1), means this lack of strength is viewed as a barrier rather than a motivator (i.e., the potential to increase muscle strength) to engagement. Similarly, a fear of falling is a commonly described barrier to RE in older people, yet reducing fall risk has been reported as a motivator to exercise [17]. Conversely, self-perceived improvements in health have been shown to promote ongoing engagement in a RE intervention [27]. Collectively, these findings highlight the need to address concerns that act as barriers for older people. Working with older adults to co-design strategies to do this that are individually tailored will be necessary to maximise impact [28].

Despite the challenges and barriers to RE engagement reported in this study, another key theme from our data was that participants expressed a willingness to perform RE under expert guidance. This is an encouraging finding and illustrates that there is potential to engage this group in RE. Our findings are consistent with Jadczak et al. [29] who reported that older adults living with frailty or prefrailty had a positive attitude to exercise. Participants in our study were keen to be provided with clear information about RE and how this exercise approach could benefit them. For older people, this information may need to come initially from a trusted source (e.g., Doctor, Primary care physician) [27] and the broader multi-disciplinary team (e.g., physiotherapist, exercise practitioners, nursing staff) have an important role to play to support initiation and ongoing engagement with a RE programme. Ensuring that older adults are active participants, as opposed to passive recipients, in the design of their exercise programme is crucial and this can help to promote engagement [30]. Adopting a flexible approach to the design and delivery of exercise programmes by embedding participant preference (e.g., individual vs. group sessions, choice of exercises, exercise location) could also help to promote engagement [31].

Our data reinforce previous work from Dekker and colleagues [32] who suggested that the design of exercise programmes for individuals living with MLTC must involve a rigorous assessment of health status and be individually adapted to specific health conditions. The patient themselves should be actively involved in these conversations [15]. Ensuring that a RE programme is tailored to an individual’s health needs (including their specific combination of MLTCs) and their specific goals should involve a range of specialists (e.g., physiotherapist, exercise practitioner, clinical exercise scientist) as well as the individual during the design stage [13]. This need for multidisciplinary teamwork is also relevant in the delivery of exercise as older adults value personal support and supervision from knowledgeable leaders during exercise sessions, providing them with reassurance and promoting self-confidence [27] which can improve adherence [33]. Informal carers and family have a role to play in promoting the role of RE as social support, from family as well as from friends or peers, has a major influence on exercise behaviour for frail older adults [22].

There remains a need for clinicians, including doctors and physiotherapists, as well as exercise practitioners to continue to challenge the persistent dogma that RE is not appropriate for older adults living with MLTC and frailty particularly those with a recent deterioration in health [34, 35]. This thinking is also illustrated by the overlapping elements of themes 1 and 2 within our data, with participants suggesting that RE is not suitable for people of their age and that age is a barrier to engagement. Resistance exercise is safe and beneficial for older people living with MLTC and frailty and should not be avoided in this group [34]. Whilst our findings have illustrated some of the specific barriers which exist in this group, further effort is needed to understand how best to overcome these barriers to enable effective implementation and delivery of RE. Older people living with MLTC and frailty must be at the heart of these conversations, along with their informal carers, family support and a range of healthcare staff (e.g., geriatricians, primary care physicians, physiotherapists, exercise practitioners).

Our findings have shown that there is a need for education to support the engagement of older people living with MLTC and frailty in RE. This strategy needs to be co-designed with older people, by formally incorporating their ideas and values into the creation and implementation of resources and services [30]. Education also needs to be targeted at family members and informal carers, clinical staff, exercise practitioners and those responsible for commissioning services. It should cover; what is RE, why RE is safe and suitable for adults living with the combination of MLTC and frailty including those with a recent deterioration in health, how to perform RE, and the potential benefits of RE. Of note, the need for RE-related education was embedded across all the themes in our data.

Finally, it is important to reinforce a key finding from our data that older adults living with MLTC, frailty and a recent deterioration in health are willing to engage in RE programmes. This finding has important implications for those designing and delivering healthcare, policy makers and those responsible for commissioning services. More work is now needed to understand how to overcome the barriers to RE identified by older adults in this study to support effective implementation of RE to this group at scale. The LiLL-OPM study [8] will provide important insights to help improve the inclusion of this group in research which will ultimately improve the evidence base for their care.

### Strengths and limitations

A major strength of this study is that the participants were recruited from an Older People’s Medicine Day Unit where research opportunities are usually limited particularly for those with a recent deterioration in health. Another strength of this work was that the interviews were conducted by a health psychologist (LD) with considerable experience in carrying out semi-structured interviews with older adults. However, it is acknowledged that our study sample, which were recruited from a single Day Unit, were predominantly female and of white British ethnicity. Older adults living with the combination of MLTC, frailty and a recent deterioration in health are a diverse group and caution is warranted when extrapolating these findings more widely.

## Conclusion

Older adults living with MLTC, frailty and a recent deterioration in health lack awareness and understanding of RE. Despite a range of barriers, this group are willing to engage in RE if they are appropriately supported. There is a need to co-design and deliver effective strategies, including education, to raise awareness and understanding of RE, as well as promote engagement in RE, in this group of older adults.

### Electronic supplementary material

Below is the link to the electronic supplementary material.

Supplementary Material 1

## Data Availability

The datasets generated and analysed during the current study are not publicly available due to participant privacy and confidentiality. De-identified data is available from the corresponding author on reasonable request.

## Abbreviations

CGA: Comprehensive Geriatric Assessment
LiLL-OPM: Lifestyle in Later Life-Older People’s Medicine
MLTC: Multiple long-term conditions
NHS: National Health Service
OPM: Older People’s Medicine
RE: Resistance exercise
SARC-F: Strength, Assistance, Rise, Climb – Falls
TA: Thematic analysis
