# Supplementary material for: Attitudes and barriers to resistance exercise training for older adults living with multiple long-term conditions, frailty, and a recent deterioration in health: qualitative findings from the Lifestyle in Later Life – Older People’s Medicine (LiLL-OPM) study
Source: BMC Geriatr. 2023 Nov 24;23:772. doi: 10.1186/s12877-023-04461-5 (PMC10675908; doi:10.1186/s12877-023-04461-5)
Supplement: Supplementary file 1 — Supplementary Material 1 [file 12877_2023_4461_MOESM1_ESM.pdf]

Additional file 1: Interview guide

Part 1: Physical activity and exercise

- How important is being physically active to you?
- What do you think the benefits / disadvantages are of being physically active?
- Currently, what might prevent you / encourage you from doing more physical activity?

- What does the term 'exercise' mean to you?
- How important is exercise to you? Why?
- What do you do now if anything that could be considered exercise? Can you describe what exercise you have done in the past?

Part 2: Resistance exercise

- Have you heard of resistance exercise?

PROMPT TO PARTICIPANT
*****Resistance exercise (or strength training) is exercise which typically involves your muscles lifting or pulling or pushing against an external weight or resistance. It often takes place in a gym and can involve lifting weights, working on machines or using elastic resistance bands*****

- Can you tell me what your thoughts are on resistance exercise?
- Do you know of any positive effects of resistance exercise?
    - How would you feel about doing this kind of exercise?
    - If you wouldn't want to do this type of exercise, why not?
    - How could we encourage and support you to engage in this type of exercise?
    - What would make you feel more comfortable about doing it?
- Have you ever been told to do more exercise by a doctor?
    - How would you feel if another professional like a physiotherapist was to advise you to exercise?
- If you had a preference for exercise at home, in hospital or in the community where would you prefer to do the exercise?
- Would you prefer to participate in group with others like yourself or individual exercise sessions? Why?
- How often do you think you could do resistance exercise (how many times per week and for how long?)
